# The three-dimensional bone mass distribution of the posterior pelvic ring and its key role in transsacral screw placement

**DOI:** 10.1038/s41598-020-61954-8

**Published:** 2020-03-30

**Authors:** Darius M. Thiesen, Dimitris Ntalos, Josephine Berger-Groch, Andreas Petersik, Bernhard Hofstätter, Karl-Heinz Frosch, Maximilian J. Hartel

**Affiliations:** 1 Department of Trauma- and Orthopaedic Surgery, University Medical Center Hamburg-Eppendorf, Martinistr. 52, 20246 Hamburg, Germany; 2 Stryker Trauma GmbH, Kiel-Schönkirchen, Germany

**Keywords:** Bone, Skeleton

## Abstract

To optimize the placement of iliosacral screws in osteoporotic bone it is essential to know where to find the best purchase. The aim of this study was to determine and visualize the distribution of bone mass in the posterior pelvic ring by using a color-coded thermal map, to differentiate the bone distribution patterns in normal pelvises and in pelvises with impaired bone density and to identify zones in S1 and S2 with particularly good bone quality, in both healthy and osteoporotic pelvises. A total of 324 pelvises were included. The bone density of the posterior pelvic ring, the fifth lumbar vertebral body (L5) and screw corridors S1 and S2 were visualized. Each individual pelvis was measured with a 3D automated program. Two groups were selected - patients with mean bone density in L5 of ≤100 HU (group 1, n = 52) and those with mean bone density >100 HU (group 2, n = 272). Color-coded thermal maps are presented of the bone density distribution in the pelvises. Bone density in L5 correlated significantly with S1 and S2; bone density was significantly higher in the S1 than in the S2 corridor (p < 0.001). Bone was denser in the posterior and upper parts of the S1 body. Bone density was significantly lower in group 2 than in group 1 (p < 0.001). The color-coded “thermal” maps of bone mass distribution can help surgeons to decide where sacroiliac screws are likely to find optimal purchase.

## Introduction

The population in industrialized countries is projected to steadily increase in age in the coming decades, with a concomitant rise in osteoporosis-related fractures^1,2^. Because of their increased prevalence, osteoporosis-related fractures of the posterior pelvic ring have received more attention over the last few years with the aim of improving treatment modalities to facilitate early mobilization and decrease mortality^3^. Only with detailed knowledge of pelvic anatomy will it be possible to understand fractures of the posterior pelvic ring and select optimal surgical treatment.

Recent imaging studies have employed 3D modelling of an osseous template and have quantified the distribution of bone mass within the posterior pelvic ring, albeit in a small patient cohort^4,5^. There were clear differences in bone density between the sacral alae and the vertebral bodies of the os sacrum. The relatively low bone mass in the paraforaminal zones is easily visible in CT scans and seems correlate with common fracture sites^3^.

There is currently no universal guideline for the treatment of osteoporosis-related fractures of the posterior pelvic ring. Moreover, there is no uniform terminology to describe these types of fractures with current terms including “insufficiency fractures”, “fatigue fractures” and “fragility fractures of the sacrum”.

Management is often conservative^6^, but immobilization in elderly patients leads to increased morbidity and is associated with two-year mortality rates of up to 49%^7,8^. Further risks of the conservative approach are non-union, secondary displacement and instability, all of which lead to persistent pain^9,10^. While surgical management is not free of complications, such as including screw loosening^9,11^, early and adequate fixation of these fractures using sacroiliac screw osteosynthesis leads to improvements in outcomes^12^.

In the current study, we aimed to determine the exact distribution of bone mass in the sacral screw corridors in the posterior pelvic ring and to differentiate bone distribution patterns in normal pelvises and in pelvises with impaired bone density. To achieve optimal screw fixation in cases of reduced bone quality, it is paramount to know which areas offer better bone purchase. A color coded map of the posterior pelvis provides more in-depth information compared to the traditional greyscale CT-slides. We hypothesized that there would be a constant lower bone density in the posterior pelvic ring screw corridors in individuals with decreased bone stock in the 5th lumbar vertebra.

## Methods

### Study population

Five hundred and thirty-seven (n = 537) raw CT datasets were analyzed. These had all originally been obtained for medical reasons: 20% patients after trauma, 70% for CT angiography and 10% for non-defined indications (median pixel spacing: 0.78 mm, median slice spacing: 1.00 mm). The data was collected retrospectively by Stryker Trauma GmbH between 2008 and 2017 with the prior written consent of the patient. Personal data such as name, date of birth or date of CT associated with the datasets were removed and not provided to the company. CT scans that included the skull were also excluded. The available demographic data generally included age, BMI and gender. In some cases, BMI, age or gender were not provided due to individual hospital data protection policies. Fractured or severely malformed pelvises (tumorous, post-osteomyelitis, post-traumatic) and pelvises with implants were excluded in advance. With these preconditions, the inclusion criteria were a fully scanned pelvis, without radiological artifacts, and the patient informed consent. The following inclusion criteria were applied to the initial study cohort: the fifth lumbar vertebra had to be included in the CT scan and the vertebral body had to be clearly defined and exhibit no degenerative changes in L5, or a sacralization or lumbalization of vertebral bodies; the S1 and S2 screw corridors (as defined below) did not interfere with the cortical boundary. This resulted in a final study cohort of 324 datasets. The mean age and standard deviation of the study population was 60.4 ± 17.6 years (17 to 93 years); 49 datasets were for patients of unknown age. There were 107 female (33%) and 217 male (67%) patients.

### 3D Modeling and analytics of bone morphology

We used SOMA, a 3D modeling and analytics software, (Stryker Orthopaedic Modeling and Analytics, Stryker GmbH, Kalamazoo, USA), which was applied to this large CT database.

**Figure 1:**
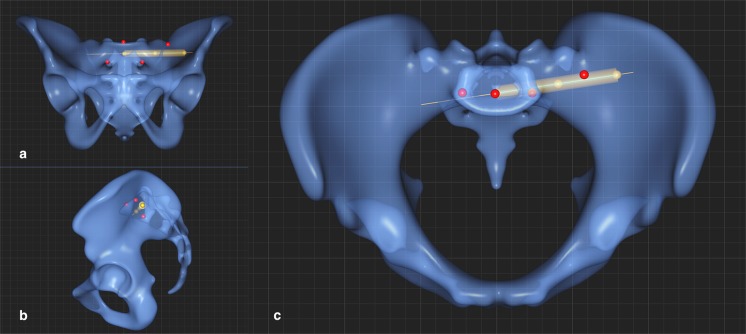
(**a**–**c**) Section a shows the definition of the left screw trajectory of S1 in the coronary plane, Section b in the sagittal plane, Section c shows the axial plane. This shows the points placed by the surgeon on the surface of the template (red points) and the points calculated by the program (orange points), i.e. in the exact center between the red points or where an imaginary line between two points intersects the surface of the templates.

**Figure 2:**
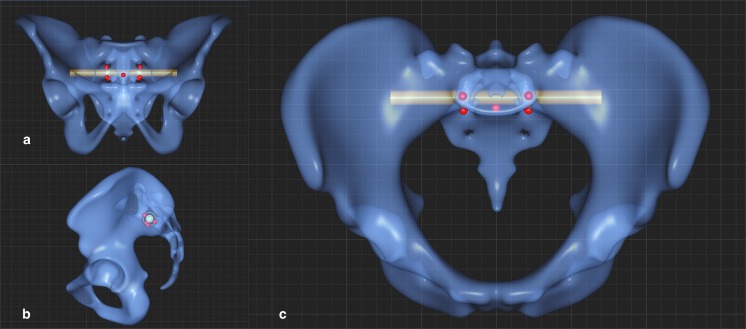
(**a**–**c**) Section a shows the definition of the screw trajectory of S2 in the coronary plane, Section b in the sagittal plane, Section c shows the axial plane. This shows where the points placed by the surgeon on the surface of the template (red points) and where the program created the calculated points (orange points), i.e. in the exact center between the red points or where an imaginary line between two points intersects the surface of the templates.

Three-dimensional models of the pelvis were created for all CT datasets by external partners using two different packages of standard segmentation software, 61% of datasets were segmented using MeVisLab, (MeVis Medical Solutions, Fraunhofer-Institut für Digitale Medizin MEVIS, Bremen, Germany) and 39% of datasets were segmented using Mimics (Materialise; Loewen, Belgium), employing a standard protocol^13^. The accuracy of the segmented bone model is determined by the Hounsfield Unit (HU) threshold outlining the boundary between bone and soft tissue. For its determination, density profiles are measured on several slices along a distance that runs completely through the dense areas of the bone to be segmented. The maximum and minimum HU values are determined for each profile and the total average is calculated from all values. The binary mask of the bone is created by an experienced operator and the threshold is applied to this binary mask upon the calculation of the 3D model. The final 3D bone model is ultimately reviewed according to the four-eyes principle. The Program can define points, lines and planes. SOMA is also able to define associated morphology and density, on the basis of a 3D pelvis template. These landmarks and measurements are applied to each of the datasets (see Figs. 1 and 2). As shown in prior studies, this method provides accurate and consistent results^13,14^. The operators who defined the bony landmarks were an experienced trauma, hip and pelvic surgeon (MH) and a trauma surgery resident (DT) with focus on pelvic trauma along with a Stryker employee (BH).

### Determination of bone density of the transiliosacral screw corridors

The screw trajectories of the first sacral vertebral corridor (S1) were defined individually for the left and right side from lateral-posterior to medial-anterior and with a slightly oblique orientation. The center of the S1 vertebral body was defined as the center point of three operator-defined points (red) on the pelvic template: one point above the middle of the vertebral body of S1, and two points at the superior margin of the left and right neuroforamina of S1 - see Fig. 1a. To define the center of the S1 corridor, an operator-defined point was positioned at the upper ventral border of the sacroiliac joint at the level of the upper plate of the S1 body. An additional point (orange) was placed between this point at the upper border of the sacroiliac joint and the point previously placed at the margin of neuroforamina S1 of the same side. This also represented the center of the S1 corridor - see too Fig. 1b. The screw trajectory for each side was then defined by the line connecting the center of the S1 vertebral body (orange) and the center of the S1 corridor (orange). Cylindrical probes representing the volume of left and right screws were defined from the entry points of the screw trajectories to the center of the S1 vertebral body, with a diameter of 10 mm.

The course of the screw trajectory through the second vertebral (S2) sacral corridor was defined in two steps. First, two points were defined to represent the center of the S2 corridor, by placing the following three points - at the lower margin of neuroforamina S1, at the upper margin of neuroforamina S2, and at the ventral cortical edge of the S2 body. In the sagittal view, a circle was defined that connects these three points, with its center defining the center of the screw corridor S2. In a second step, a point was positioned in the middle of the S2 body at the ventral cortical edge (red) and, in order to avoid cutting through the ventral cortex of S2, the final screw corridor was defined so that it runs at least 7 mm dorsally from this point. The cylindrical probe was defined by the left and right entry points of the screw trajectory and with a diameter of 10 mm; see also Fig. 2.

In a further step, different areas of interest - such as the vertebral bodies of S1 and S2 and the ala - were investigated separately. To this end, and to ensure statistical evaluation of the data, the left and right S1 probes were divided into 1% steps relative to their combined length and the same procedure was followed for the length of the S2 probe. The mean bone density in the vertebral bodies was measured from 40% to 60% of the total probe length. In the same way, bone density was measured for the alae in the ranges 20–40% and 60–80% - with respect to total probe length - and mean bone density was calculated from the combined left and right sides.

After defining the probes, the dataset of a total of 537 pelvises was subjected to a manual optical check to see whether all corridors lay within the cortical boundaries. Due to differences in sacral morphology and/or sacral- or lumbalization of the vertebral body, 175 pelvises were excluded. The measurement of bone density was performed with SOMA for each of 324 individual pelvises and expressed in Hounsfield Units for segments of 0.25 mm of the probes.

### Determination of the bone density of the fifth lumbar body

Studies have shown that bone density measured in Hounsfield Units (HU) of the lumbar spine clearly correlates with bone mineral density in the transverse and sagittal CT views^15,16^. For this reason and because L5 is the closest vertebral body to the posterior pelvic ring and is routinely included in CT studies of the pelvis, patients were distinguished by the average bone density of L5.

The measurement of bone density in the fifth lumbar vertebral body (L5) was performed manually with Mimics® Software 21.0 (Materialise; Loewen, Belgium). To establish the precise mean bone density of the fifth vertebral body, a volume was individually defined within the cortical borders of L5 for each of 324 patients. Using sagittal views, the measuring volume was positioned manually in the middle of L5 of the trabecular bone^15^ and placed in the middle of the vertebral body using axial views, adapted to lumbar lordosis^16^, so that the maximum trabecular mass without cortical involvement was included in the measurement.

After determining the exact mean bone density of L5, two groups were assembled: patients with mean bone density of >100 HU (n = 272) “group 1”, and patients with a mean bone density of ≤ 100 HU (n = 52) - “group 2”. The threshold of 100 HU was chosen on the basis of recent studies^17^ that indicate that this value is very specific for the diagnosis of osteoporosis from CT scans. The mean values and standard deviations of bone density were determined in Hounsfield Units.

**Figure 3:**
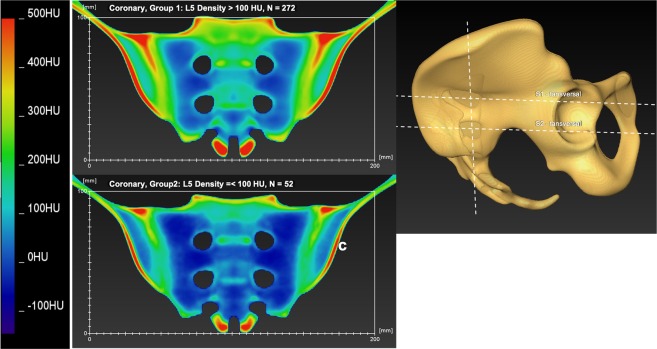
(**a**–**c**) Shows a thermal map of the bone density of the coronary plane of the calculated model of the average pelvis in groups 1 and 2, as defined by the bone density of L5. Section a represents group 1, Section b shows group 2 and Section c gives an image of the position of the coronary plane.

**Figure 4:**
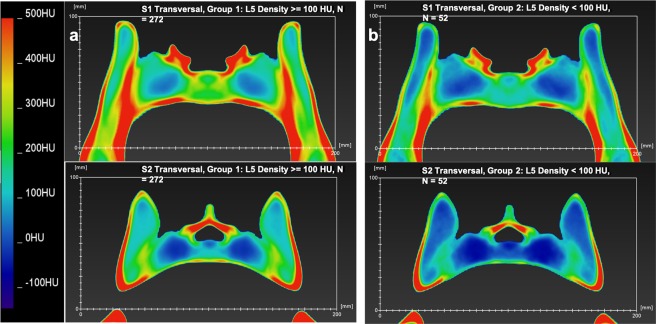
(**a**,**b**) Shows a thermal map of the bone density of the axial plane of the calculated model of the average pelvis at the level of the S1 and S2 corridor of group 1 and 2 defined by the bone density of L5. Section a represents group 1, Section b shows group 2.

To visualize the mean density distribution of the pelvis, SOMA was used to calculate mean bone density models for the two groups defined by the L5 mean density of ≤100 HU (n = 52) and >100 HU (n = 272). Cross sectional images showing color-coded density distribution were created using FEI Amira 6.3.0. In these images, the denser the bone, the “hotter” it appears on the color-coded (thermal) map. This means that blue areas have a lower bone density and are followed in ascending order by yellow, green and red areas. See results section, Figs. 3 and 4.

## Statistics

Continuous parameters are reported as means. Categorical variables are reported as absolute counts with percentages in brackets. We used the Shapiro-Wilk test to evaluate the normal distribution of the data. Non-parametric methods were employed for the statistical analyses of variables with non-normal distribution. We used Spearman’s rho for the calculation of correlations between continuous variables. The Wilcoxon test was carried out to compare continuous variables between groups. Multiple linear regression analyses were used to determine the relative predictive power of the linear correlation of metric variables - such as age, sex and mean bone density of L5 - with the total variance of bone density. This was carried out for both screw corridors and for the vertebral bodies, the entire course of the corridors and the alae. The level of significance for all statistical tests was 95% (alpha = 0.05). The Statistical analyses were all carried out by using IBM SPSS V21 (IBM Corp., NY, USA).

All methods were conducted in accordance with relevant guidelines and regulations. This study has been evaluated and authorized by our local ethics committee of “Aerztekammer Hamburg”, WF -087/18.

## Results

For an overview of the bone mass distribution, see Figs. 3 and 4 below. The bone mass of L5, S1 and S2 in Hounsfield Units correlated significantly with age in both sexes. For women, the correlation between age and bone mass in the vertebral body was closer than for men (female L5: r = 0.667; S1: r = 0.67; S2: r = 0.679). For all pelvises, a significant correlation was found between the mean bone mass of L5 and the mean bone mass of the screw corridor of S1 and S2 (S1: r = 0.84; S2: r = 0.79).

**Figure 5:**
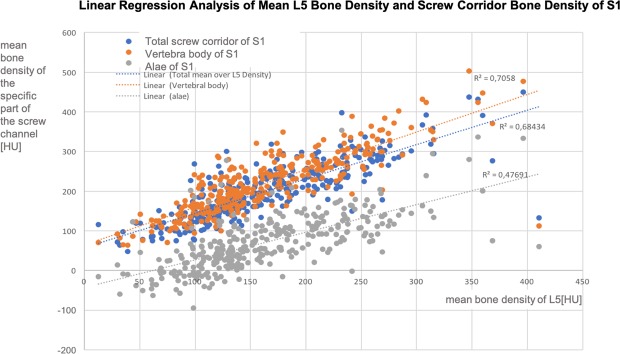
Depicts the linear regression analysis of the mean bone density of L5 (Hounsfield Units; x-axis) with specific parts of the bone density in the screw channels (Hounsfield Units; y-axis); orange = vertebral body of S1, blue = overall screw channel bone density of S1 and grey = sacral alae of S1.

A linear regression analysis demonstrated that the bone density of L5 explains 71% of the variance of the bone density in the vertebral body of S1, 68% of bone density in the whole S1 corridor and 48% of the bone density in the alae at the S1 corridor level, respectively (p < 0.001, adjusted r^2^ = 0.705, r^2^ = 0.684, r^2^ = 0.477); see Fig. 5.

A linear regression analysis demonstrated that the bone density of L5 explains 61% of the variance of the bone density in the vertebral body of S2, 64% of bone density in the whole S2 corridor and 42% of the bone density in the alae at the S2 corridor level, respectively.

Gender was not a significant predictor of L5 bone density, whereas age was (p < 0.001, r^2^ = 0.321).

**Figure 6:**
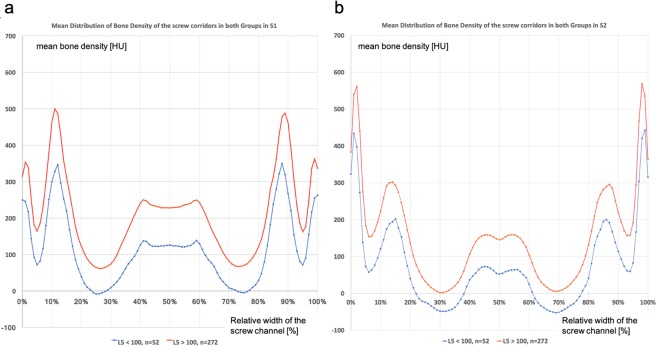
(**a**,**b**) Section a shows the results for the S1 corridor, Section b for the S2 corridor. In both graphs, the x-axis shows the relative percentage of the width of each pelvis, while the y-axis represents bone density in Hounsfield Units. The red dots and line represent group 1 (HU of L5 > 100); the blue dots and line represent group 2 (HU of L5 ≤ 100).

The mean bone density of the S1 corridor, vertebral body and sacral alae was significantly elevated than that of the S2 corridors (p < 0.001). The relative proportion of female individuals and the average age was significantly higher in Group 2 (L5 bone density ≤100 HU) compared to the total population (group 1 = 57 ± 17.3 y; group 2 = 75 ± 9 y; p < 0.001). The average bone density of the 5^th^ lumbar vertebra was 179 ± 60 HU for group 1 and 76 ± 22 HU for group 2 (p < 0.001). The bone density of the vertebral bodies of S1, S2, the alae and the entire screw corridor in group 2 was significantly less than in group 1 (p < 0.001), see also Table 1 and Fig. 6.

**Table 1:** Gives an overview of the different groups by age and mean bone density of the L5 vertebral body.

	Number	Age [y]	Female Patients	Bone Density L5 [HU]	Bone Density Corridor S1 [HU]	Bone Density Vertebral Body S1 [HU]	Bone Density Sacral Alae Level S1 [HU]	Bone Density Corridor S2 [HU]	Bone Density Vertebral Body S2 [HU]	Bone Density Sacral Alae Level S2 [HU]
		Mean ± SD	Number (%)	Mean ± SD (range)	Mean ± SD (range)	Mean ± SD (range)	Mean ± SD (range)	Mean ± SD (range)	Mean ± SD (range)	Mean ± SD (range)
**Total Population**	324	60.4 ± 17.6	107 (33)	163 ± 68 (12–410)	198 ± 71 (48–449)	218 ± 77 (63–503)	70 ± 69 (−96–357)	143 ± 60 (23–322)	133 ± 69 (−14–347)	30 ± 55 (−83–208)
**L5 > 100 HU**	272	57 ± 17.3	83 (31)	179.1 ± 60 (101–410)	213 ± 64 (99–449)	236 ± 69 (99–503)	82 ± 64 (−44–357)	157 ± 55 (50–323)	147 ± 63 (8–347)	41 ± 51 (−64–208)
**L5 ≤ 100 HU**	52	75.5 ± 9	24 (46)	76 ± 22 (13–100)	114 ± 38 (48–268)	126 ± 35 (63–243)	8 ± 56 (−96–205)	72 ± 30 (23–176)	58 ± 42 (−15–239)	−23 ± 41 (−83–146)
***p-value***	*—*	<*0.001*	*0.03*	<*0.001*	<*0.001*	<*0.001*	<*0.001*	<*0.001*	<*0.001*	<*0.001*
**<60 y**	126	44.3 ± 11	40 (32)	199 ± 62 (35–396)	237 ± 67 (96–449)	256 ± 67 (65–503)	101 ± 66 (−29–357)	179 ± 56 (19–347)	169 ± 66 (19–347)	55 ± 51 (−52–208)
**≥60 y**	150	73.9 ± 8	56 (37)	135 ± 63 (13–410)	167 ± 60 (48–357)	192 ± 70 (63–401)	47 ± 62 (−96–296)	114 ± 48 (23–256)	105 ± 58 (−15–299)	9 ± 48 (−83–146)
***p-value***		<*0.001*	*0.33*	<*0.001*	<*0.001*	<*0.001*	<*0.001*	<*0.001*	<*0.001*	<*0.001*

This also gives an overview of the demographics of our population. In the section right of the thick lines, the overall bone density of the whole corridor and specific parts such as vertebral body and sacral alae are listed for S1 and S2.

*SD* = *standard deviation; HU* = *Hounsfield Units; y* = *years*.

We prepared two diagrams to show the average bone density of the screw corridor of S1 and S2 and its connection to the pelvic, see Fig. 6. The x-axis shows the relative percentage of the width of each pelvis, while the y-axis represents bone density in Hounsfield Units. The two peaks on both sides show the os ilium cortex; the second peaks, starting at the side, represent the sacroiliac joint and its cortices at 15% and 85% of the pelvic width. Further towards the middle of the pelvis, a clear decrease in bone density can be seen, reflecting the bilateral alae void from 20 to 40% and from 80 to 60% of pelvic width. The bone density then increases again. This increase is attributed to the vertebral bodies of S1 and S2. For the S1 vertebral body, the bone density shows a plateau, in contrast to a hill shaped distribution for S2. Across the entire width, each mean of group 1 (L5 > 100 HU) shows a higher bone density than group 2 (L5 ≤ 100 HU).

## Discussion

The color-coded thermal maps give the surgeon a precise and visually appealing display of the bone density distribution. The measurements of the screw channels S1 and S2 showed a clear pattern of bone density distribution, with a high density at the cortical parts, i.e. the os ilium and the sacroiliac joint, and a much lower, or even nominally negative, bone density at the alae of the sacrum. Overall, the bone density in the vertebral bodies of S1 and S2 were significantly different, with a lower value in S2, which confirms the results of Wagner *et al*.^5^. Therefore, it appears that S1 assures better screw purchase than S2.

When patients were classified by the bone density of the L5 vertebra, there was a significant difference in mean bone density between the entire corridor of S1 and S2, the vertebral bodies and the sacral alae, which confirmed our hypothesis. As known from clinical practice and previously demonstrated by a 3D measurement of 91 pelvises^5^ and a cadaver study^18^, the paraforaminal zone or alar void, was confirmed, while the bone density of L5 was the strongest indicator of its expression. Our findings of low Hounsfield Units in the paraforaminal region from S1 extending to S3 may explain the typical fracture pattern in osteoporotic-related fractures of the pelvis^19,20^. In most cases, the fracture line is located in the Denis zone I, but horizontal fracture lines running through the S2 vertebral body can also be observed in up to one fifth of all fractures^12^, which is consistent with our findings of significantly lower bone density distribution in the S2 corridor and in the vertebral body.

The largest difference in bone density between the two groups was found in the vertebral bodies, which is of particular importance as this is the location where the screws are placed and where their thread ensures their retention. This observation might explain screw loosening in osteoporotic patients without bilateral cortical fixation^9,21,22^. To circumvent the obstacle of low cancellous bone quality in the vertebral bodies, complete transsacral fixation with longer screws or bars might be used^10,23^. These implants are based on cortical bone quality and seem to offer more biomechanical stability in osteoporotic bone, as calculated by an infinite model^24,25^.

In a biomechanical cadaver study, the combination of a locked transsacral screw and one iliosacral screw showed better results than two “standard” iliosacral screws^26^, suggesting that the highest stability can be achieved when the thread passes through the contralateral sacroiliac joint and the os ilium cortex. However, in their biomechanical cadaver study, Salari *et al*.^27^ found no significant difference in stability between transsacral fixation and a long iliosacral screw, where this long screw did not perforate the contralateral sacroiliac joint. The latter biomechanical study seems to support our conclusion that there is a wide zone of sufficient bone quality in the vertebral bodies, which provides purchase similar to that of the cortex of the contralateral sacrum^27^. Some surgeons avoid fixation of the contralateral, uninjured sacroiliac joint in order to prevent pain, stiffness and delays in rehabilitation. However, these concerns seem to have been dispelled by Heydemann *et al*.^28^, who examined 53 patients after posterior pelvic fixation using transsacral or iliosacral techniques and found no differences in pain or functional values after one year. In their study, only younger individuals (mean age 41 years) were included, so no direct comparison with elderly patients is possible. It is currently unclear whether complete transsacral screw fixation or iliosacral fixation in osteoporotic fractures is superior in a clinical setting.; This might be a topic for future research.

Closer examination of the produced thermal map of the bone density distribution in the individual vertebral bodies of S1 and S2 may also provide valuable insights. In S2, the bone mass in the anterior third and upper part of the vertebral body was denser; but, in the S1 vertebral body, the bone density of the cancellous bone was surprisingly lower in the anterior third than in the middle part, but considerably higher in the upper part of the vertebral body; see Figs. 3 and 4. The optimal placement of transiliosacral screws is thought to be at the anterior part of the S1 vertebral body, since biomechanical studies have shown that the highest thread tension is in this area^11^ and retrospective analysis of screw placement in the S1 body has shown a significantly higher loosening rate of screws in the center of the vertebral body than with screws placed in the anterior part^29^. The latter study seems to contradict our findings for bone density distribution in S1, but Kim *et al*.^29^ defined screw placement by analyzing postoperative inlet radiographs images that tend not to be as accurate as CT images, which may have affected their results. However, it may be that higher screw purchase is often ensured by fixation of the screws in the anterior cortex and not by pure cancellous bone fixation^30^. In healthy bone, better screw fixation could be achieved by placing the thread in the upper central portion of the S1 vertebra, as shown in the thermal map, Fig. 3. The exact role of screw loosening and cortical contact may be the subject of future biomechanical research.

A limitation of our study was the type of CT scans we examined. Since there was no quantitative calibration to bone mass (qCT), an exact diagnosis of osteoporosis or osteopenia was not possible. Another point of criticism could be that the scans were performed with different CT scanners from different manufacturers. While this could have influenced the results, it must be noted that all scans were performed with similar high-quality technical specifications, with high resolution and thin slices. It is also currently unclear whether osteoporosis can be diagnosed by measuring Hounsfield units alone via CT^15^. This could be another drawback of our study, as there was no dual-energy X-ray absorption (DXA) measurement available in our cohort. Therefore, the definition of “osteoporosis” from the HU values (as used in this paper) may be inaccurate. Nevertheless, this procedure is used in clinical practice and has been shown to be reasonably accurate in the diagnosis of osteoporosis or osteopenia, as described by Pickhardt *et al*. with an AUC of 0.83 to 0.864^16,17^.

In conclusion, a color-coded heat map of the pelvis showing the bone mass distribution may be of practical value for surgeons in making decisions on implant placement.

It was shown that the bone mass of the posterior pelvic ring highly correlated with the bone mass of the vertebral body of L5 and that the characteristic fracture morphology in osteoporosis-related fractures were confirmed by our 3D model.

The vertebral body of S1 showed an area of high bone density in the posterior and upper part, which is contrary to clinical experience, since the best screw purchase is typically achieved in the anterior third.
